# A prospect on the use of antiviral drugs to control local outbreaks of COVID-19

**DOI:** 10.1186/s12916-020-01636-4

**Published:** 2020-06-25

**Authors:** Andrea Torneri, Pieter Libin, Joris Vanderlocht, Anne-Mieke Vandamme, Johan Neyts, Niel Hens

**Affiliations:** 1 Centre for Health Economic Research and Modelling Infectious Diseases, University of Antwerp, Antwerp, Belgium; 2 Interuniversity Institute of Biostatistics and Statistical Bioinformatics, Data Science Institute, Hasselt University, Hasselt, Belgium; 3 Artificial Intelligence Lab, Department of Computer Science, Vrije Universiteit Brussel, Brussels, Belgium; 4 Department of Microbiology and Immunology, Rega Institute for Medical Research, Clinical and Epidemiological Virology, KU Leuven - University of Leuven, Leuven, Belgium; 5 Center for Global Health and Tropical Medicine, Unidade de Microbiologia, Instituto de Higiene e Medicina Tropical, Universidade Nova de Lisboa, Lisbon, Portugal

## Abstract

**Background:**

Current outbreaks of COVID-19 are threatening the health care systems of several countries around the world. Control measures, based on isolation, contact tracing, and quarantine, can decrease and delay the burden of the ongoing epidemic. With respect to the ongoing COVID-19 epidemic, recent modeling work shows that these interventions may be inadequate to control local outbreaks, even when perfect isolation is assumed. The effect of infectiousness prior to symptom onset combined with asymptomatic infectees further complicates the use of contact tracing. We aim to study whether antivirals, which decrease the viral load and reduce infectiousness, could be integrated into control measures in order to augment the feasibility of controlling the epidemic.

**Methods:**

Using a simulation-based model of viral transmission, we tested the efficacy of different intervention measures to control local COVID-19 outbreaks. For individuals that were identified through contact tracing, we evaluate two procedures: monitoring individuals for symptoms onset and testing of individuals. Additionally, we investigate the implementation of an antiviral compound combined with the contact tracing process.

**Results:**

For an infectious disease in which asymptomatic and presymptomatic infections are plausible, an intervention measure based on contact tracing performs better when combined with testing instead of monitoring, provided that the test is able to detect infections during the incubation period. Antiviral drugs, in combination with contact tracing, quarantine, and isolation, result in a significant decrease of the final size and the peak incidence, and increase the probability that the outbreak will fade out.

**Conclusion:**

In all tested scenarios, the model highlights the benefits of control measures based on the testing of traced individuals. In addition, the administration of an antiviral drug, together with quarantine, isolation, and contact tracing, is shown to decrease the spread of the epidemic. This control measure could be an effective strategy to control local and re-emerging outbreaks of COVID-19.

## Background

The use of invasive non-pharmaceutical interventions (i.e. full city lockdown (Wuhan), school closures, cutting inter-city travel and intra-city mobility) was able to bring the epidemic under control in China [1, 2], but these measures are associated with profound societal and economic disruptions. We investigate the use of contact tracing and isolation in combination with an antiviral compound to control local outbreaks of COVID-19, to avoid such invasive social measures, and to preemptively reduce the burden of the epidemic. Even when perfect isolation is in place, this may not be sufficient to contain a local COVID-19 outbreak [3], due to presymptomatic transmission [4, 5]. Therefore, in the absence of a vaccine, an antiviral drug in addition to isolation could be used to contain the current COVID-19 epidemic. There are currently no potent and selective antivirals available against coronaviruses. The development of such potent and safe drugs typically takes 10 years or longer. However, there are a number of drugs that either directly target a viral enzyme (such as the viral RNA-dependent RNA polymerase, e.g., remdesivir and favipiravir) or that have been developed for non-viral indications, but that exert at least some level of antiviral activity (the so-called repurposed drugs). We here assume that an antiviral drug will reduce the viral load of an infected individual with COVID-19. For our modeling experiments, we considered the experimental drug remdesivir, for which viral load data were available to inform our model. Remdesivir is an investigational, broad-spectrum antiviral agent that was developed for the treatment of Ebola virus infections. It is a nucleotide analog that inhibits the viral RNA-dependent RNA polymerase and has activity against a wide range of RNA viruses [6]. It is also active against SARS-CoV and MERS-CoV, which can be explained by similarities in the active site of the polymerase of these viruses. Based on this promising activity against other coronaviruses, remdesivir was recently shown to also inhibit SARS-CoV-2 in vitro [7]. As a consequence, this drug is currently under evaluation against COVID-19 in various clinical trials, and based on preliminary efficacy data, the drug was granted emergency use designation for severe COVID-19 patients by the FDA on 1 May 2020. For the aforementioned reasons, we chose to inform our model with data on the control of MERS-CoV viral load by remdesivir in a translational murine model [8]. This animal model was specifically developed to better approximate the pharmacokinetics and drug exposure profile in humans. Therefore, the measure of viral titers in lung tissue at different time points in this murine model serves as a reasonable proxy for viral dynamics upon compound exposure in the controlled setting of a viral challenge. To this end, we calibrate the model to represent the viral load decrease thereof.

In this manuscript, we first present the effect of isolation, considering both home quarantine (for individuals that are part of a contact trace network and for infected individuals with mild symptoms) and hospital isolation (for severe cases). We argue that when an individual is quarantined at home, this will only result in a partial reduction of contacts, since contacts with household members remain and other breaks of isolation can occur. To compensate for this imperfect isolation, we consider the use of an antiviral compound. We test these different control measures in a simulation study that aims at representing, given the available information, the current COVID-19 epidemic. Many countries are already beyond the point where local containment alone will suffice. However, we do expect that the methodology we propose will be key to avoid a second peak, especially given the limited depletion of susceptibles.

## Methods

### Epidemiological dynamics

**Fig. 1:**
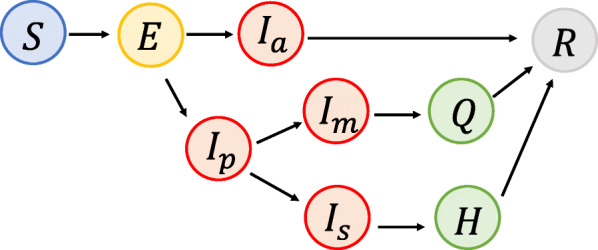
Disease dynamics. Possible transitions among the different epidemic compartments

The disease dynamics are depicted in the left panel of Fig. 1. The possible transitions between epidemic classes are described by the arrows.

Individuals are initially susceptible (*S*), and once infected, they enter the exposed class (*E*). The infection can be asymptomatic (*I*_*a*_) if individuals do not show symptoms during their infectious periods or symptomatic. Symptomatic individuals, after a presymptomatic period (*I*_*p*_), can show mild (*I*_*m*_) or severe symptoms (*I*_*s*_). When diagnosed, symptomatic individuals are hospitalized (*H*) or are confined in home quarantine (*Q*), based on the severity of symptoms. We assumed that hospitalized individuals are immediately isolated. Asymptomatic individuals, however, are assumed to not be diagnosed. Ultimately, all infectives are assumed to either recover from infection or die (*R*). Isolation and quarantine start at the time of diagnosis. Isolation is assumed to be perfect; therefore, individuals can no longer transmit the disease. The quarantined individuals, instead, can still make contacts, although at a decreased rate.

### Transmission model

The transition from the susceptible to the exposed class is governed by a stochastic process based on the notion of infectious contact processes [9]. First, contacts between individuals are generated. When such contacts are generated between susceptible and infectious people, these can result in an infection event according to a Bernoulli experiment, using a probability value based on the time since infection. This probability is computed, at a precise time point, as the product of two components: the infectiousness measure, *υ*(*t*), which quantifies the level of infectiousness over time, and the total amount of infectivity *q*, i.e., the number of expected effective contacts over the contact rate [10]. The function *υ*(*t*) is defined over the exposed and infectious period, or analogously over the incubation and symptomatic period, along which it integrates to one. This function is scaled to have a similar shape among different infectives, based on their lengths of exposed and infectious period. According to this framework, an infectious individual makes effective contacts at a rate, *r*(*t*), given by:

1 $$ r(t)=\lambda \times q\times \upsilon (t), $$

where *λ* is the contact rate. The mean number of effective contacts is an approximation of the basic reproduction number. The two quantities are identical in an infinite and homogeneous population, where the probability that an individual makes two effective contacts with the same person is zero. For the considered population size, the likelihood that this event occurs is extremely low. Therefore, throughout the manuscript, we approximate the basic reproduction number with the mean number of effective contacts.

In this framework, isolation and quarantine are implemented by reducing the contact rate *λ*. The infectiousness measure *υ*(*t*) is set to represent the shape of the viral load curve according to the assumption that higher viral load corresponds to higher transmission probability.

### Simulation parameters and distributions

In Table 1, we report the parameters and distributions that were utilized in the simulation study. Where distributions are not reported, the parameters are assumed to be constant. In the last column, we report the literature references that justify our choice of parameter value—or distribution—we used.

**Table 1:** Model parameters

Name	Mean value (SD)	Distribution	Reference
Incubation period	5.2 days (2.8 days)	Weibull	[11, 12]
Symptomatic period length	18 days	Exponential	[13]
Time to diagnosis	3.8 days (2.45 days)	Gamma	[14]
Basic reproduction number			
Symptomatic ${\mathcal{R}}_0^s$	2.5	NA	[11, 12, 15, 16]
Asymptomatic ${\mathcal{R}}_0^a$	1.375	NA	[17]
Symptomatic Individuals	69%	NA	[18]
Severe cases	16%	NA	[19]
Population size	1000	NA	Motivated in the text
Daily contact rate	12 contacts	NA	[20]
Infectiousness measure	10 days (3.8 days)	Gamma	[13, 21, 22]

We assume that symptomatic individuals make, on average, between two and three effective contacts. This value is set accordingly to the current estimate of the basic reproduction number [11, 12, 15, 16]. The infectiousness measure is set to represent the viral load observations reported in [13, 21, 22], the peak of which is reached within a few days after symptom onset. Based on the currently available information, we assume the same viral load shape between mild, severe, and asymptomatic individuals. The total length is informed using the observations of symptomatic cases reported in Zou et al. [13], and it is computed as the convolution of incubation and symptomatic period. Asymptomatic individuals are assumed to have lower transmission compared to symptomatic ones. We set the asymptomatic reproduction number, ${\mathcal{R}}_0^a$, to be equal to 0.55 ${\mathcal{R}}_0^s$. This value was selected from the estimation presented in Li et al. [17] for unreported cases. The proportion of asymptomatic individuals is set to be equal to 31% [18].

The time to diagnosis is assumed to coincide with the time of hospitalization. We set this value in line with the estimation reported in Donnelly et al. [14] for SARS. The chosen value is in line with recent estimation of the time to hospitalization for COVID-19 reported in Linton et al. [23]. At the time of diagnosis, symptomatic individuals, depending on the severity of the symptoms, are isolated (severe cases) or quarantined (mild cases). The population size is set to 1000 to represent a localized outbreak of COVID-19. We assume the population to be homogeneous, closed, and finite. These assumptions relate to the control measures currently in place, which aim at containing immigration to and emigration from a country with an ongoing outbreak.

### Contact tracing and isolation

In order to implement contact tracing, we keep track of a contact history ${\mathcal{H}}_i$ for each individual *i,* for all contacts made since the time of infection. When an individual *i* is found to be infected with SARS-CoV-2, a contact tracing procedure is started. We assume that each contact in ${\mathcal{H}}_i$ will be traced back successfully with probability *η*. Depending on the considered scenario, traced back individuals will be monitored or tested, using a PCR test. When individuals are found positive for SARS-CoV-2, they will be put in quarantine/isolation. For certain scenarios, next to quarantine, we also inject infected individuals with an antiviral drug (i.e., remdesivir).

We assume that the PCR test can detect infection after 2 days since infection. Traced individuals who test negative the first time are tested again after 2 days. The quarantine will result in a decreased contact rate (i.e., imperfect isolation), *λ*_*q*_, while in case of perfect isolation the contact rate is set to zero. Similarly, diagnosed individuals will also be quarantined: at home (mild symptoms), with a decreased contact rate *λ*_*q*_, or in the hospital (severe symptoms), where we assume that perfect isolation is possible. In all the considered scenarios, 16% of individuals are isolated while the rest are placed in quarantine. The selected proportion reflects the proportion of severe cases reported in Guan et al. [19]

### Antiviral compounds

**Fig. 2:**
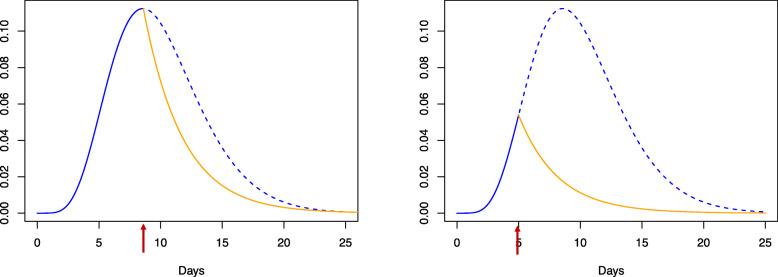
Reduction of infectiousness. The blue and the orange lines describe the infectiousness measure, respectively, before (dashed blue) and after (solid yellow) antiviral administration. The red arrows indicate the start of the antiviral treatment (i.e., remdesivir)

To compensate for imperfect isolation, we investigate the use of antiviral compounds to reduce the infectiousness of an infected individual. Following earlier work [24], we assume that once the antiviral compound has been administered, the infectiousness measure will exponentially decay according to an inverse Malthusian growth model (shown in Fig. 2) [25]. The rate of this decay is set to represent the reduction in viral load, due to remdesevir, as reported in [8] for the MERS coronavirus.

### Scenarios

We assume the following parameters for the reduction of contacts because of home quarantine: *λ*_*q*_ = 0.1*λ*, 0.25*λ*, 0.5*λ* and for the probability of tracing back a contact in history ${\mathcal{H}}_i$: *η* = 0.25, 0.5, 0.75 [26].

In all the considered scenarios, we assume that individuals are isolated, or quarantined, when diagnosed. Moreover, we assume that contact tracing starts at the time of diagnosis.

- *IAS*. Traced individuals are monitored for 2 weeks and isolated/quarantined if they show symptoms during this period. This scenario is similar to the baseline scenario described by Hellewell et al. [3] with the exception that in our description only severe cases are isolated while the mild cases are home quarantined. In addition, we include a proportion of asymptomatic individuals that are not detected. This scenario better reflects the current practice of containment.
- *IBS*. Traced individuals are isolated/quarantined, as soon as they test positive for SARS-COV-2. We assume that the PCR test can detect a positive individual 2 days after infection. Therefore, a traced individual is tested immediately when traced, and if this test was negative, we test the individual again 2 days later.
- *IBTBS*. A diagnosed patient is immediately treated with the antiviral drug. Furthermore, traced individuals are isolated/quarantined and injected with the antiviral drug, as soon as they test positive for SARS-COV-2. We assume that the PCR test can detect a positive individual 2 days after infection. Therefore, a traced individual is tested immediately when traced, and if this test was negative, we test the individual again 2 days later.

For each scenario, we run 5000 simulations. Among these, we compute the final size and the cases at peak for the one in which at least the 10% of individuals have been infected. Doing this, we only account for outbreaks that are most challenging to contain.

## Results

**Fig. 3:**
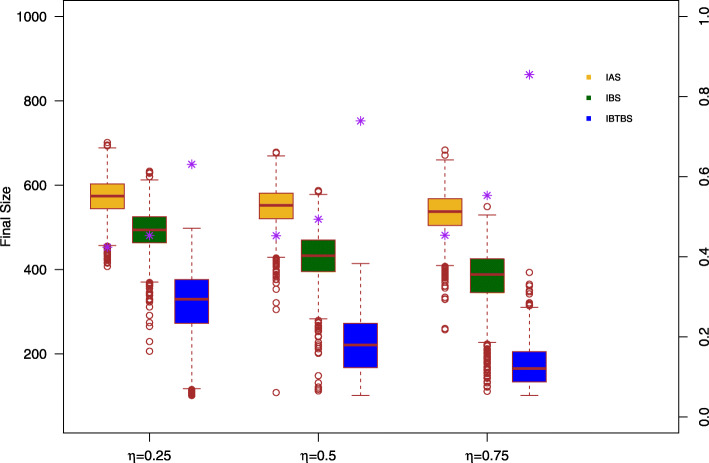
Final size distribution. Distributions of the final size value for scenario IAS (yellow), scenario IBS (green), and scenario IBTBS (blue) when the quarantine contact rate is λ_q_ = 0.25λ together with the probability that a simulation leads to a number of cases smaller than the 10% of the population (purple asterisks)

**Fig. 4:**
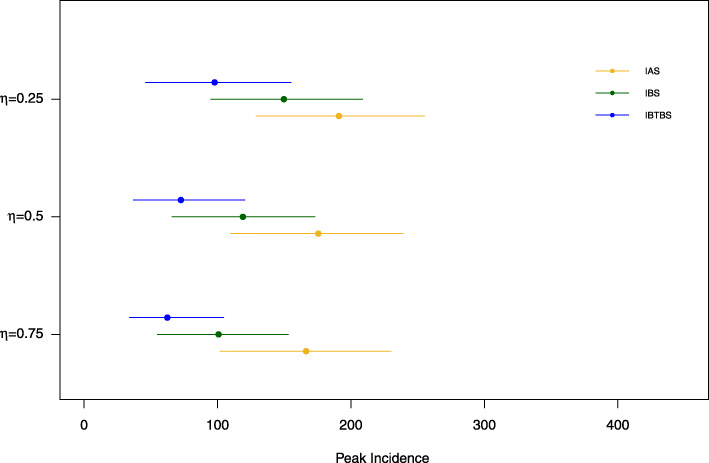
Peak incidence. Mean peak incidence value for scenario IAS (yellow), scenario IBS (green), and scenario IBTBS (blue) together with the 2.5% and the 97.5% percentiles

Quarantine, isolation, and antiviral treatments lead, in different levels, to the mitigation of the outbreak by reducing the final size as well as by reducing the number of cases at the peak of the epidemic. A control measure based on monitoring is less effective in containing the epidemic, even when a high proportion of contacts are successfully traced. Instead, when a test is performed on the traced individuals, the mitigation efficacy increases depending on the probability of successfully traced contacts (Figs. 3 and 4) since asymptomatic individuals are possibly detected and the traced individuals who test positive are immediately quarantined or isolated limiting presymptomatic and asymptomatic infections. Isolation and quarantine are therefore more effective when performed prior to symptom onset, which is important, as recent work shows that infectees are infectious prior to symptom onset [5].

In terms of the magnitude among intervention strategies, the antiviral treatment is shown to have the larger impact and, together with quarantine and isolation, significantly reduces the final size, the peak incidence, and the number of outbreaks that are most challenging to contain.

## Discussion

In this modeling study, a number of assumptions were made, which we here discuss.

We assume an exponential decay model for the infectiousness when the antiviral compound is administered. To challenge this assumption, we perform a sensitivity analysis where we consider a logistic curve (i.e., the Gompertz model [27]: another common way to model biological population processes) and a linear function (Additional File 1: Fig. S1). This sensitivity analysis shows that the different decay models yield similar results, supporting the robustness of our proposed prevention scheme (Additional File 1: Fig. S2).

Regarding transmissibility, we tested the effect of a longer mean incubation period of 6.4 days [28], while keeping the infectiousness measure profile fixed. Doing so, we investigated a higher probability of presymptomatic infections. Results reported in Additional File 1: Fig. S6 indicate similar performance of the control strategies based on testing, while monitoring is shown to be less effective. Since SARS-CoV-2 also spreads in the presymptomatic stage, testing and administering antivirals are expected to be beneficial to reduce the number of infectives.

In our baseline scenario, we assume that infected individuals can be identified using PCR testing starting 2 days post-infection. Furthermore, we investigated the effect of a test that can only detect infection after 3 and 4 days since infection (Additional File 1: Fig. S11 and Fig. S12). As expected, the efficacy of a control measure based on testing is sensitive to detection timeframes, but the control measure that uses antiviral drugs is still the most effective among the ones that were tested. Nevertheless, it is important to note that early diagnosis seems feasible based on this recent work [29], where individuals in a nursing facility were found positive through PCR test, prior to symptom onset. This indicates that it is possible to diagnose patients early after infection. Another recently published paper [4] states that infectious coronavirus particles are shed at high concentrations from the nasal cavity before symptom development, which again indicates that it could be possible to detect infection early by means of PCR test.

We assume that we have sufficient antiviral drug doses to treat all individuals that are encountered via the contact tracing procedure and enough tests to detect their infectious status. This is motivated by the fact that we consider an emerging outbreak and the required number of doses and tests will thus be limited.

Furthermore, in the baseline scenario, we assume that all symptomatic individuals are in fact diagnosed. Due to the awareness of COVID-19 given by media and government officials, individuals are more likely to act upon even mild symptoms. This assumption is in line with the work of Hellewell et al. [3]. While this is a limitation of our study, we argue that in the scenarios where we aggressively trace and treat the contacts of individuals, we are more likely to detect (and constrain) cases that would otherwise go undetected. In addition, we investigated the impact of a proportion of mild symptomatic individuals that are not being diagnosed, unless they are traced (Additional File 1: Fig. S3, Fig. S4, and Fig. S5). As expected, all the tested control measures perform better when a high percentage of individuals are diagnosed. However, the use of antivirals in combination with testing and isolation/quarantine remarkably reduces the total number of cases and the peak incidence also in these scenarios.

When severe cases are diagnosed or test positive, we assume they are immediately placed in perfect isolation, meaning that these infected individuals can no longer transmit the infection. While this assumption is consistent with earlier work [3], health care workers are still at risk as they could be infected by infected individuals being cared for in isolation.

In the IBS and IBTBS scenarios, we assume that the traced individuals that test positive are isolated in 16% of cases, even before showing actual severe symptoms.

Although this model is informed with data on the control of MERS-CoV viral load using prophylaxis with remdesivir, it stands to reason that different classes of viral inhibitors control the viral load in different ways. Additionally, despite the sequence similarity of MERS-CoV and SARS-COV-2, it remains to be established whether the impact of remdesivir (or other antivirals) on the viral load is similar. Recently, clinical trials addressing the use of remdesivir in the treatment of SARS-CoV-2 patients in different phases of their disease course were initiated. The clinical outcomes of such antiviral administrations are starting to be published in literature, where the starting time of the treatment seems to be crucial [30, 31]. Wang et al. reported a randomized trial in which no statistically significant benefit was associated with remdesivir [31]. However, it has been argued that this trial is underpowered and therefore results in inconclusive findings [32]. In addition, in that trial, the median time before the start of treatment was 10 days from the start of disease onset, possibly reflecting a phase in which the efficacy of antiviral administration is more limited. In this phase, not only the viral presence but also an excessive inflammatory response is a major cause of patient death [33, 34]. Moreover, a more positive statement on the efficacy of remdesivir was recently released, in which a moderate clinical improvement in recovery time was reported in an additional clinical trial (NCT04292899) [35].

However, the relationship between the effect of the antiviral on the viral load is not available yet, especially if administered in the early stage of the infection. To this end, longitudinal data of the viral load on COVID-19 infected patients treated with different viral inhibitors will be informative, especially when the administration is performed in the early stage of infection. Furthermore, Sheahan et al. [8] demonstrated that the efficacy of remdesivir for MERS depends on the viral dose and also on the timing of the treatment of the viral inhibitor. When mice were inoculated with a high inoculum of virus, delayed start of antiviral treatment failed to fully prevent viral pathogenesis. Although remdesivir proved effective at reducing the viral load also under these conditions, the argument for an early start of antiviral treatment is evident. Presumably, reducing the viral load with an antiviral compound loses its efficacy in advanced disease as the tissue damage is sustained by inflammatory processes in absence of the viral initiator. We here suggest to consider the proposed scenario based on remdesivir. In our implementation, antiviral injections are immediately administered to successfully traced individuals, mostly in their asymptomatic phase, and to the diagnosed patient. Therefore, we believe that the assumptions on the use of this drug, in the considered scenario, are reasonable. Furthermore, as remdesivir is a repurposed drug that was developed for Ebola, it remains possible that more potent coronavirus inhibitors will be developed in the future.

We considered a homogeneous population that lacks structured contact patterns. In fact, the probability of successfully tracing contacts depends on the mixing behavior of the specific individual. In particular, close contacts within the household are likely to be traced as well as contacts in working places, school, and leisure environments [36]. Since the majority of close contacts occur in these locations, it is reasonable to assume that a high proportion of secondary infections can be correctly traced. Therefore, this analysis should be further investigated in a more fine-grained individual based model prior to moving this strategy to public health practice.

While a limited number of countries (Singapore, Hong Kong, South Korea) were able to control the epidemic through contact tracing without the use of an antiviral compound, many countries (e.g., Italy, France, Spain, Belgium, USA) so far failed using this approach, partly due to limited capacity to implement similar measures. Therefore, we believe an approach that can accommodate contract tracing imperfections, e.g., by reducing infectiousness through the use of an antiviral compound, is warranted.

We also tested the impact of outbreaks that are initiated by multiple infectives, and the results are shown in Additional File 1: Fig. S16. Final size and peak incidence approximate the baseline scenario but the fade-out probability decreases. Nevertheless, the administration of antivirals in combination with the other measures still results in a higher fade-out probability compared to the other tested scenarios, reducing the probability that an outbreak will be major.

## Conclusion

The ongoing epidemic of COVID-19 threatens to overwhelm the health systems of many countries. Although control measures such as isolation and quarantine are important, their exclusive use may not be able to efficiently contain an outbreak, requiring a prolonged lockdown that could harm the economic system. In addition, when a high proportion of infected individuals require hospital care, the number of cases at peak should be minimized as much as possible to avoid regional health care infrastructure becoming overwhelmed. In the current study, we highlight the importance of testing compared to monitoring, and we show the impact of an antiviral compound that reduces the viral load and, consequently, the infectiousness of infectives. For this simulation study, we utilized the data on the control of the viral load by remdesivir in a pathogen challenge experiment. However, this study can be easily extended to other antivirals, given the availability of viral load data upon drug administration. Although the efficacy of administering an antiviral compound, in addition to isolation and quarantine, depends on the effectiveness of the respective drug, it is plausible that drugs that interfere with the infection life cycle show a comparable impact on the viral load. We demonstrate that the implementation of such a compound together with quarantine leads to a substantial reduction of the final size and the peak incidence. In addition, the number of outbreaks that are most challenging to contain decreases when the antiviral is administered to diagnosed and traced individuals. The same result holds when the basic reproduction number is set to an equal value for symptomatic and asymptomatic individuals (Additional File 1: Fig. S11) and when the proportion of asymptomatic and diagnosed individuals vary among the population (Additional File 1: Fig. S3, Fig. S4, Fig. S5, Fig. S14, and Fig. S15). Therefore, the administration of an antiviral drug, together with isolation and quarantine, is expected to have a major impact in the control of local COVID-19 outbreaks. Although antiviral drugs are mostly evaluated in clinical trials for their capacity to control the disease manifestations and alter the clinical outcome in individual patients, our study implies that antiviral drugs can be implemented to alter the spread of a viral epidemic by influencing the viral load and infectiousness of infectees upon quarantine. Given the fact that antiviral treatment should be given in the quarantine setting, the route of administration in relation to the frequency and duration of treatment and the safety of the compound in healthy volunteers are critical for the success of such an intervention. Although oral formulations are desirable, intraveneous application in a limited outbreak could prevent hospitalizations and thus reduce the pressure on the health care capacity as the antiviral treatment is initiated earlier in the disease.

However, such a treatment falls in many countries under the responsibility of a prescribing practitioner that is typically not part of the track and contact trace team.

An important consideration is the use of contact tracing in combination with an antiviral to reduce the burden on nursing home facilities. Recent work shows that despite lockdowns, SARS-CoV-2 continues to spread in such facilities, resulting in a high number of deaths [29]. We believe that the implementation of frequent testing, contact tracing in combination with pharmaceutical intervention (with an antiviral) to treat individuals of a nursing home facility early, could have an important impact on the number of deaths in these facilities.

We remain hopeful for drugs to be identified, either directly or indirectly targeting the virus, that can be used as a treatment for the hospitalized patient population. Yet, our work shows that such compounds have an additional potential to mitigate the pandemic when implemented in concert with quarantine and contact tracing. Therefore, research aimed at the identification of new drugs and drug formulations suitable for the prophylactic use for different viral families with pandemic potential remains warranted.

## Supplementary information

**Additional file 1.** Sensitivity Analysis.

## Data Availability

Source code of the simulation model is available in the GitHub repository at the following link: https://github.com/AndreaTorneri/ViralTransm. The simulation code was developed using the software R.

## References

[1] Tian H, Liu Y, Li Y, Wu C-H, Chen B, Kraemer MU, Li B, Cai J, Xu B, Yang Q, et al. An investigation of transmission control measures during the first 50 days of the COVID-19 epidemic in China. Science. 2020;368(6491):638-42. 10.1126/science.abb6105 PMC7164389 32234804

[2] Kraemer MU, Yang C-H, Gutierrez B, Wu C-H, Klein B, Pigott DM, du Plessis L, Faria NR, Li R, Hanage WP, et al. The effect of human mobility and control measures on the COVID-19 epidemic in China. Science. 2020;368(6490):493-97. 10.1126/science.abb4218 PMC7146642 32213647

[3] Hellewell J, Abbott S, Gimma A, Bosse NI, Jarvis CI, Russell TW, Munday JD, Kucharski AJ, Edmunds WJ, Sun F, et al. Feasibility of controlling COVID-19 outbreaks by isolation of cases and contacts. Lancet Glob Health. 2020;8(4):e488-96. 10.1016/S2214-109X(20)30074-7 PMC7097845 32119825

[4] Gandhi M, Yokoe DS, Havlir DV. Asymptomatic transmission, the Achilles’ heel of current strategies to control Covid-19. N Engl J Med. 2020. 10.1056/NEJMe2009758. 10.1056/NEJMe2009758 PMC7200054 32329972

[5] Ganyani T, Kremer C, Chen D, Torneri A, Faes C, Wallinga J, Hens N. Estimating the generation interval for coronavirus disease (COVID-19) based on symptom onset data, March 2020. Eurosurveillance. 2020;25(17). 10.2807/1560-7917.ES.2020.25.17.2000257 PMC7201952 32372755

[6] Warren TK, Jordan R, Lo MK, Ray AS, Mackman RL, Soloveva V, Siegel D, Perron M, Bannister R, Hui HC (2016). Therapeutic efficacy of the small molecule gs-5734 against Ebola virus in rhesus monkeys. Nature.

[7] Wang M, Cao R, Zhang L, Yang X, Liu J, Xu M, Shi Z, Hu Z, Zhong W, Xiao G (2020). Remdesivir and chloroquine effectively inhibit the recently emerged novel coronavirus (2019-ncov) in vitro. Cell Res.

[8] Sheahan, T.P., Sims, A.C., Leist, S.R., Sch¨afer, A., Won, J., Brown, A.J., Montgomery, S.A., Hogg, A., Babusis, D., Clarke, M.O., Spahn, J.E., Bauer, L., Sellers, S., Porter, D., Feng, J.Y., Cihlar, T., Jordan, R., Denison, M.R., Baric, R.S.: Comparative therapeutic efficacy of remdesivir and combination lopinavir, ritonavir, and interferon beta against mers-cov. Nature Communications 11(1), 222 (2020). 10.1038/s41467-019-13940-6 PMC6954302 31924756

[9] Rhodes PH, Halloran ME, Longini IM (1996). Counting process models for infectious disease data: distinguishing exposure to infection from susceptibility. J R Stat Soc Ser B Methodol.

[10] Svensson °A. A note on generation times in epidemic models. Mathematical biosciences. 2007;208(1):300–11. 10.1016/j.mbs.2006.10.010 17174352

[11] Zhang J, Litvinova M, Wang W, Wang Y, Deng X, Chen X, Li M, Zheng W, Yi L, Chen X, Wu Q, Liang Y, Wang X, Yang J, Sun K, Longini IM, Halloran ME, Wu P, Cowling BJ, Merler S, Viboud C, Vespignani A, Ajelli M, Yu H. Evolving epidemiology of novel coronavirus diseases 2019 and possible interruption of local transmission outside Hubei province in China: a descriptive and modeling study. The Lancet Infectious Diseases. 2020. 10.1016/S1473-3099(20)30230-9. 10.1016/S1473-3099(20)30230-9 PMC7269887 32247326

[12] Li Q, Guan X, Wu, P, Wang X, Zhou L, Tong Y, Ren R, Leung KSM, Lau EHY, Wong JY, Xing X, Xiang N, Wu Y, Li C, Chen Q, Li D, Liu T, Zhao J, Liu M, Tu W, Chen C, Jin L, Yang R, Wang Q, Zhou S, Wang R, Liu H, Luo Y, Liu Y, Shao G, Li H, Tao Z, Yang Y, Deng Z, Liu B, Ma Z, Zhang Y, Shi G, Lam TTY, Wu JT, Gao GF, Cowling BJ, Yang B, Leung GM, Feng Z. Early transmission dynamics in Wuhan, China, of Novel Coronavirus - Infected Pneumonia. New England J Med. 2020;382(13):1199-207. 10.1056/NEJMoa2001316 PMC7121484 31995857

[13] Zou L, Ruan F, Huang M, Liang L, Huang H, Hong Z, Yu J, Kang M, Song Y, Xia J, et al. SARS-CoV-2 viral load in upper respiratory specimens of infected patients. N Engl J Med. 2020;382(12):1177-9. 10.1056/NEJMc2001737 PMC7121626 32074444

[14] Donnelly CA, Ghani AC, Leung GM, Hedley AJ, Fraser C, Riley S, Abu-Raddad LJ, Ho L-M, Thach T-Q, Chau P, Chan K-P, Lam T-H, Tse L-Y, Tsang T, Liu S-H, Kong JH, Lau EM, Ferguson NM, Anderson RM (2003). Epidemiological determinants of spread of causal agent of severe acute respiratory syndrome in Hong Kong. Lancet.

[15] Flahault A. Has China faced only a herald wave of SARS-CoV-2? The Lancet. 2020;395(10228):947. 10.1016/S0140-6736(20)30521-3 PMC7124610 32145187

[16] World Health Organization: Coronavirus disease 2019 - (COVID19). situation report - 46. https://www.who.int/docs/default-source/coronaviruse/situation-reports/20200306-sitrep-46-covid-19.pdf?sfvrsn=96b04adf2.

[17] Li R, Pei S, Chen B, Song Y, Zhang T, Yang W, Shaman J. Substantial undocumented infection facilitates the rapid dissemination of novel coronavirus (SARS-CoV-2). Science. 2020;368(6490):489-93. 10.1126/science.abb3221 PMC7164387 32179701

[18] Nishiura H, Kobayashi T, Suzuki A, Katsuma Hayashi S-MJ, Kinoshita R, Yang Y, Yuan B, Akhmetzhanov AR, Linton NM. Estimation of the asymptomatic ratio of novel coronavirus infections (covid-19). Int J Infect Dis. 2020. 10.1016/j.ijid.2020.03.020 PMC7270890 32179137

[19] Guan, W.-j., Ni, Z.-y., Hu, Y., Liang, W.-h., Ou, C.-q., He, J.-x., Liu, L., Shan, H., Lei, C.-l., Hui, D.S.C., Du, B., Li, L.-j., Zeng, G., Yuen, K.-Y., Chen, R.-c., Tang, C.-l., Wang, T., Chen, P.-y., Xiang, J., Li, S.-y., Wang, J.-l., Liang, Z.-j., Peng, Y.-x., Wei, L., Liu, Y., Hu, Y.-h., Peng, P., Wang, J.-m., Liu, J.-y., Chen, Z., Li, G., Zheng, Z.-j., Qiu, S.-q., Luo, J., Ye, C.-j., Zhu, S.-y., Zhong, N.-s.: Clinical characteristics of coronavirus disease 2019 in china. New England J Med 2020. 10.1056/NEJMoa2002032. 10.1056/NEJMoa2002032 PMC7092819 32109013

[20] Willem L, Hoang TV, Funk S, Coletti P, Beutels P, Hens N. Socrates: An online tool leveraging a social contact data sharing initiative to assess mitigation strategies for covid-19. medRxiv. 2020. 10.1101/2020.03.03.20030627. 10.1186/s13104-020-05136-9 PMC7296890 32546245

[21] Zhou P, Yang X-L, Wang X-G, Hu B, Zhang L, Zhang W, Si H-R, Zhu Y, Li B, Huang C-L, Chen H-D, Chen J, Luo Y, Guo H, Jiang R-D, Liu M-Q, Chen Y, Shen X-R, Wang X, Zheng X-S, Zhao K, Chen Q-J, Deng F, Liu L-L, Yan B, Zhan F-X, Wang Y-Y, Xiao G-F, Shi Z-L. A pneumonia outbreak associated with a new coronavirus of probable bat origin. Nature. 2020. 10.1038/s41586-0202012-7.

[22] Pan Y, Zhang D, Yang P, Poon LLM, Wang Q. Viral load of SARS-CoV-2 in clinical samples. The Lancet Infectious Diseases. 2020;20(4):411-2. 10.1016/S1473-3099(20)30113-4 PMC7128099 32105638

[23] Linton N, Kobayashi T, Yang Y, Hayashi K, Akhmetzhanov A, Jung S-M, Yuan B, Kinoshita R, Nishiura H (2020). Incubation period and other epidemiological characteristics of 2019 novel coronavirus infections with right truncation: a statistical analysis of publicly available case data. J Clin Med.

[24] Bonhoeffer S, May RM, Shaw GM, Nowak MA (1997). Virus dynamics and drug therapy. Proc Natl Acad Sci.

[25] Malthus TR, Winch D, James P. Malthus: an essay on the principle of population. Cambridge University Press. 1992.

[26] Klinkenberg, D., Fraser, C., Heesterbeek, H.: The effectiveness of contact tracing in emerging epidemics. PloS one 1(1) (2006). 10.1371/journal.pone.0000012 PMC1762362 17183638

[27] Gompertz, B.: Xxiv. On the nature of the function expressive of the law of human mortality, and on a new mode of determining the value of life contingencies. in a letter to francis baily, esq. frs &c. Philosophical transactions of the Royal Society of London (115), 513–583 (1825). 10.1098/rstb.2014.0379 PMC4360127 25750242

[28] Backer JA, Klinkenberg D, Wallinga J. Incubation period of 2019 novel coronavirus (2019-ncov) infections among travellers from Wuhan, China, 20–28 january 2020. Eurosurveillance. 2020;25(5). 10.2807/1560-7917.ES.2020.25.5.2000062 PMC7014672 32046819

[29] Arons MM, Hatfield KM, Reddy SC, Kimball A, James A, Jacobs JR, Taylor J, Spicer K, Bardossy AC, Oakley LP, et al. Presymptomatic SARS-CoV-2 infections and transmission in a skilled nursing facility. N Engl J Med. 2020. doi: https://doi.org/10.1056/NEJMoa2008457. 10.1056/NEJMoa2008457 PMC7200056 32329971

[30] Grein J, Ohmagari N, Shin D, Diaz G, Asperges E, Castagna A, Feldt T, Green G, Green ML, Lescure F-X, Nicastri E, Oda R, Yo K, Quiros-Roldan E, Studemeister A, Redinski J, Ahmed S, Bernett J, Chelliah D, Chen D, Chihara S, Cohen SH, Cunningham J, D’Arminio Monforte A, Ismail S, Kato H, Lapadula G, L’Her E, Maeno T, Majumder S, Massari M, Mora-Rillo M, Mutoh Y, Nguyen D, Verweij E, Zoufaly A, Osinusi AO, DeZure A, Zhao Y, Zhong L, Chokkalingam A, Elboudwarej E, Telep L, Timbs L, Henne I, Sellers S, Cao H, Tan SK, Winterbourne L, Desai P, Mera R, Gaggar A, Myers RP, Brainard DM, Childs R, Flanigan T. Compassionate use of remdesivir for patients with severe covid-19. New England J Med. 2020. 10.1056/NEJMoa2007016.

[31] Wang, Y., Zhang, D., Du, G., Du, R., Zhao, J., Jin, Y., Fu, S., Gao, L., Cheng, Z., Lu, Q., Hu, Y., Luo, G., Wang, K., Lu, Y., Li, H., Wang, S., Ruan, S., Yang, C., Mei, C., Wang, Y., Ding, D., Wu, F., Tang, X., Ye, X., Ye, Y., Liu, B., Yang, J., Yin, W., Wang, A., Fan, G., Zhou, F., Liu, Z., Gu, X., Xu, J., Shang, L., Zhang, Y., Cao, L., Guo, T., Wan, Y., Qin, H., Jiang, Y., Jaki, T., Hayden, F.G., Horby, P.W., Cao, B., Wang, C.: Remdesivir in adults with severe COVID-19: a randomised, double-blind, placebo-controlled, multicentre trial. The Lancet 2020; 395(10236):1569-1578. 10.1016/S0140-6736(20)31022-9 PMC7190303 32423584

[32] Norrie JD. Remdesivir for COVID-19: challenges of underpowered studies. Lancet. 2020. 10.1016/S0140-6736(20)31023-0. 10.1016/S0140-6736(20)31023-0 PMC7190306 32423580

[33] Merad M, Martin JC. Pathological inflammation in patients with COVID-19: a key role for monocytes and macrophages. Nat Rev Immunol. 2020. 10.1038/s41577-020-0331-4. 10.1038/s41577-020-0331-4 PMC7201395 32376901

[34] Richardson S, Hirsch JS, Narasimhan M, et al. Presenting characteristics, comorbidities, and outcomes among 5700 patients hospitalized with COVID-19 in the New York City area. JAMA Published online April 22, 2020. doi:10.1001/jama.2020.6775. 10.1001/jama.2020.6775 PMC7177629 32320003

[35] Remdesivir shows modest benefit in coronavirus trial. (2020). New York Times https://www.nytimes.com/2020/04/29/health/gilead-remdesivir-coronavirus.html. Accessed 13 May 2020.

[36] Mossong J, Hens N, Jit M, Beutels P, Auranen K, Mikolajczyk R, Massari M, Salmaso S, Tomba GS, Wallinga J, Heijne J, SadkowskaTodys M, Rosinska M, Edmunds WJ (2008). Social contacts and mixing patterns relevant to the spread of infectious diseases. PLoS Med.

