# Supplementary material for: A prospect on the use of antiviral drugs to control local outbreaks of COVID-19
Source: BMC Med. 2020 Jun 25;18:191. doi: 10.1186/s12916-020-01636-4 (PMC7315692; doi:10.1186/s12916-020-01636-4)
Supplement: Supplementary file 1 — Additional file 1. Sensitivity Analysis. [file 12916_2020_1636_MOESM1_ESM.pdf]

Sensitivity Analysis

In this section we report the sensitivity analysis in which the following quantities are tested:

- Decay rates
- Proportion of diagnosed individuals
- Mean incubation period length
- Quarantine contact rate
- Reproduction number
- Test detection period
- Asymptomatic proportion
- Initial Infectives

Decay rates

We tested different decay rates to model the effect of the antiviral drug on the viral load and consequently on the infectiousness measure. Together with the exponential rate presented in the baseline scenario, we model the decay rate via a sigmoid function, using a Gompertz model [1], and via a constant function. As shown in Figure S1, we set these models to have the same values after 4 days, representing the decrease in viral load induced by Remdesevir in as study on MERS [2]. Final size and peak incidence are not affected by the tested decay rates as shown in Figure S2.

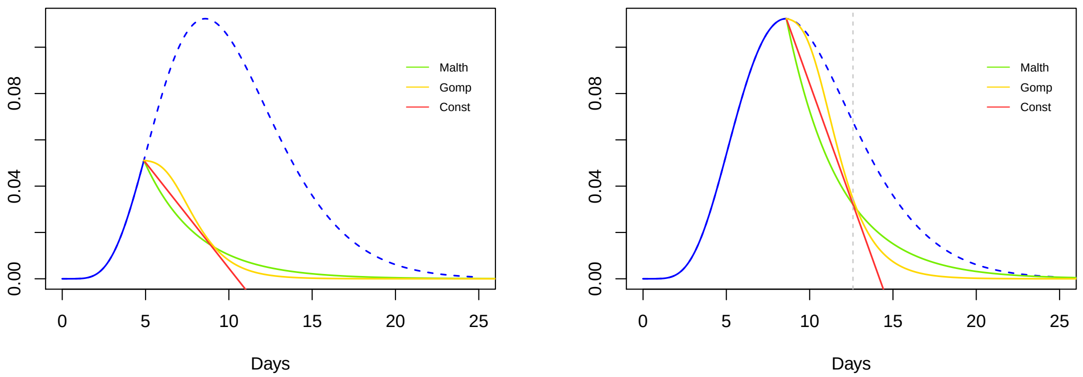

Figure S1. Decay rates of infectiousness measure after the administration of an antiviral: Malthusian (green), Gompertz (yellow) and constant (red).

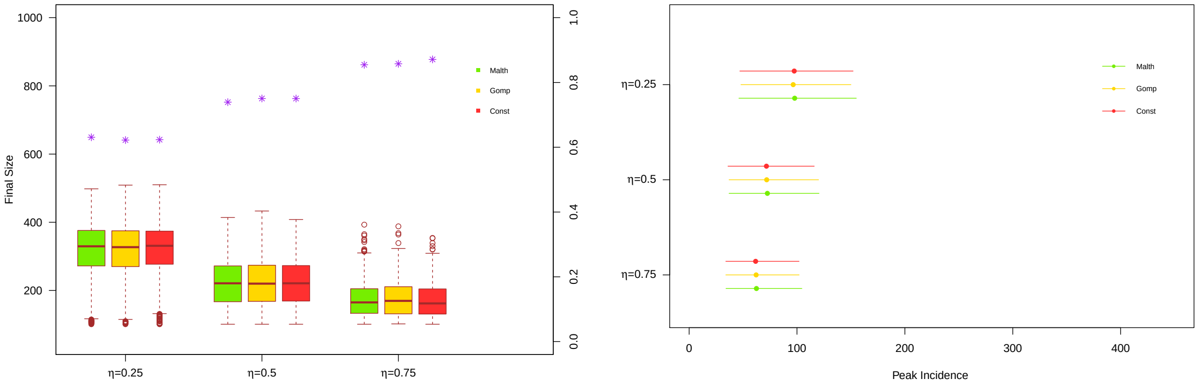

Figure S2. Final size distribution (left panel) and mean peak incidence (right panel) when the decay rate is described by a Matlhusian, a Gompertz or a linear model. The quarantine contact rate is λ_q=0.25λ, $\mathcal{R}_0^s = 2.5$ and $\mathcal{R}_0^a = 0.55\mathcal{R}_0^s$. In the left panel, for each scenario we report the probability that a simulation leads to a number of cases smaller than the 10% of the population (purple asterisks). In the right panel, together with the point estimates we report the 2.5% and 97.5 % percentiles.

Proportion of diagnosed individuals

We assume that only mild cases can escape diagnoses, when not identified via contact tracing. We vary the proportion of diagnosed symptomatic individuals that is here tested to be: 25%, 50% and 75%. As in the baseline scenario, asymptomatic individuals can be diagnosed only when traced and tested. The effect on the final size and peak incidence are reported, respectively, in Figure S3, Figure S4 and Figure S5 . As expected, these summary measures increase when less symptomatic individuals are diagnosed. However, among all the tested proportions the control strategy based on antivirals, contact tracing and quarantine/isolation results always to be the best approach to decrease final size and peak incidence.

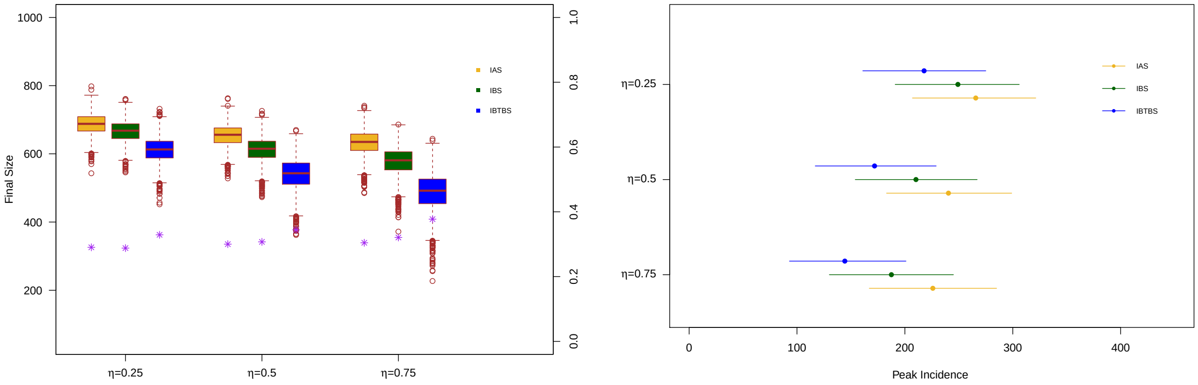

Figure S3. Final size distribution (left panel) and mean peak incidence (right panel) for Scenario IAS (yellow), Scenario IBS (green) and Scenario IBTBS (blue) when the proportion of diagnosed (mild) symptomatic individuals is 25%. The quarantine contact rate is set to λ_q=0.25 λ, and the reproduction numbers are $\mathcal{R}_0^s = 2.5$,$\mathcal{R}_0^a$ =0.55$\mathcal{R}_0^s$. In the left panel, for each scenario we report the probability that a simulation leads to a number of cases smaller than the 10% of the population (purple asterisks). In the right panel, together with the point estimates we report the 2.5% and 97.5 % percentiles.

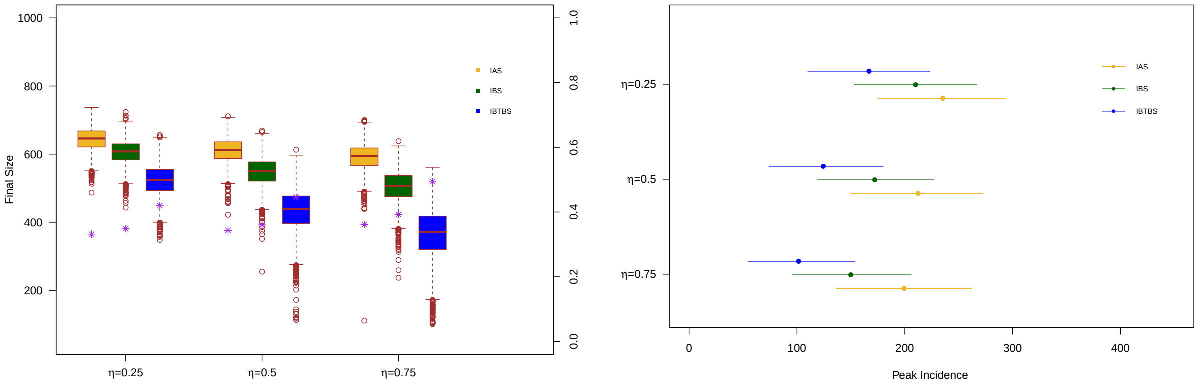

Figure S4. Final size distribution (left panel) and mean peak incidence (right panel) for Scenario IAS (yellow), Scenario IBS (green) and Scenario IBTBS (blue) when the proportion of diagnosed (mild) symptomatic individuals is 50%. The quarantine contact rate is set to λ_q=0,25 λ, and the reproduction numbers are $\mathcal{R}_0^s = 2.5$,$\mathcal{R}_0^a$ =0.55$\mathcal{R}_0^s$. In the left panel, for each scenario we report the probability that a simulation leads to a number of cases smaller than the 10% of the population (purple asterisks). In the right panel, together with the point estimates we report the 2.5% and 97.5 % percentiles.

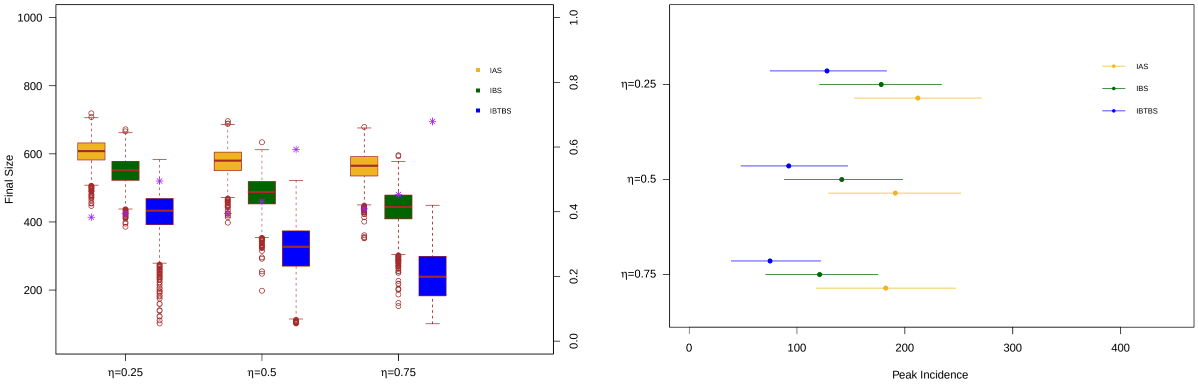

Figure S5. Final size distribution (left panel) and mean peak incidence (right panel) for Scenario IAS (yellow), Scenario IBS (green) and Scenario IBTBS (blue) when the proportion of diagnosed (mild) symptomatic individuals is 75%. The quarantine contact rate is set to λ_q=0.25 λ, and the reproduction numbers are $\mathcal{R}_0^s = 2.5$,$\mathcal{R}_0^a$ =0.55$\mathcal{R}_0^s$ .In the left panel, for each scenario we report the probability that a simulation leads to a number of cases smaller than the 10% of the population (purple asterisks). In the right panel, together with the point estimates we report the 2.5% and 97.5 % percentiles.

Mean incubation period length

We test the mean length of the incubation period setting this value to 6.4 days, in line with the findings reported in Backer et al. [3]. The resulting infectiousness measure presents a longer presymptomatic phase. To this regard, final size and peak incidence result slightly higher for the IAS scenario, where traced individuals are only monitored (Figure S6) thus spreading in the presymptomatic period. For the other two scenario instead, since infectives are similarly identified in the presymptomatic phase, results are approximately the same as the ones in the baseline scenario (Figure 3 and 4 result section).

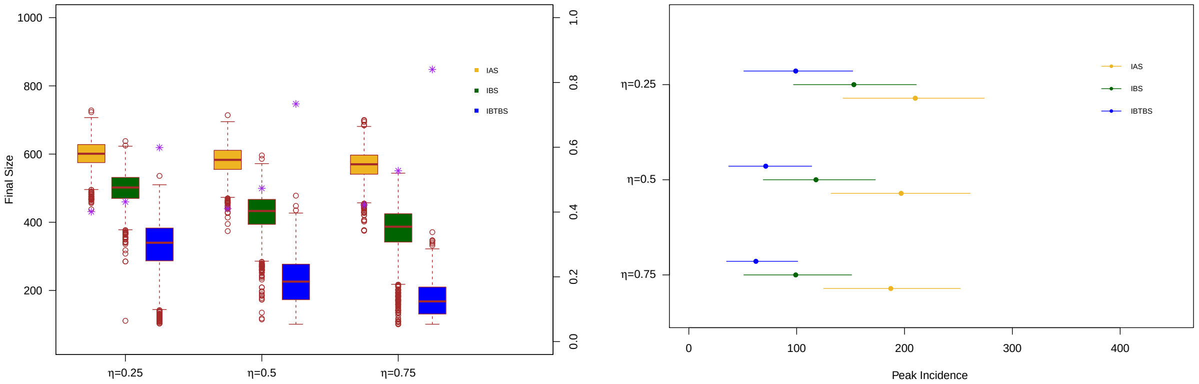

Figure S6. Final size distribution (left panel) and mean peak incidence (right panel) for Scenario IAS (yellow), Scenario IBS (green) and Scenario IBTBS (blue) when the expected incubation period length is 6.4 days. The quarantine contact rate is λ_q=0,25λ, $\mathcal{R}_0^s = 2.5$ and $\mathcal{R}_0^a = 0.55\mathcal{R}_0^s$. In the left panel, for each scenario we report the probability that a simulation leads to a number of cases smaller than the 10% of the population (purple asterisks). In the right panel, together with the point estimates we report the 2.5% and 97.5 % percentiles.

Quarantine contact rate

We report in Figure S7 and S8 the sensitivity analysis for the quarantine contact rate: $\lambda_q = 0.1\lambda, 0.5\lambda$. The introduction of an antiviral compound substantially contributes in reducing the final size, the peak incidence and the probability of a challenging outbreak in all the considered settings. This decrease, compared to the scenario in which only isolation/quarantine is implemented, increases when quarantine is less effective (left panels).

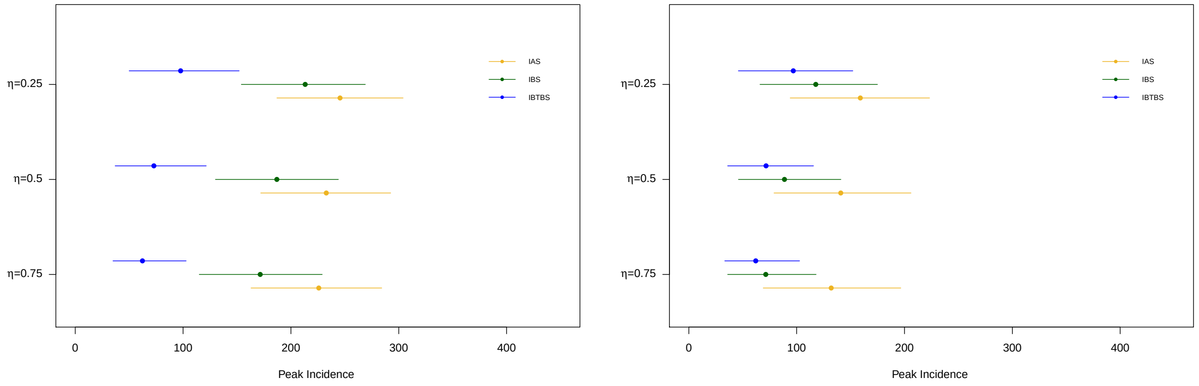

Figure S7. Mean peak incidence for Scenario IAS (yellow), Scenario IBS (green) and Scenario IBTBS (blue) when the quarantine contact rate is λ_q=0.5λ (left panel) and λ_q=0.1λ (right panel) together with 2.5% and 97.5% percentiles.

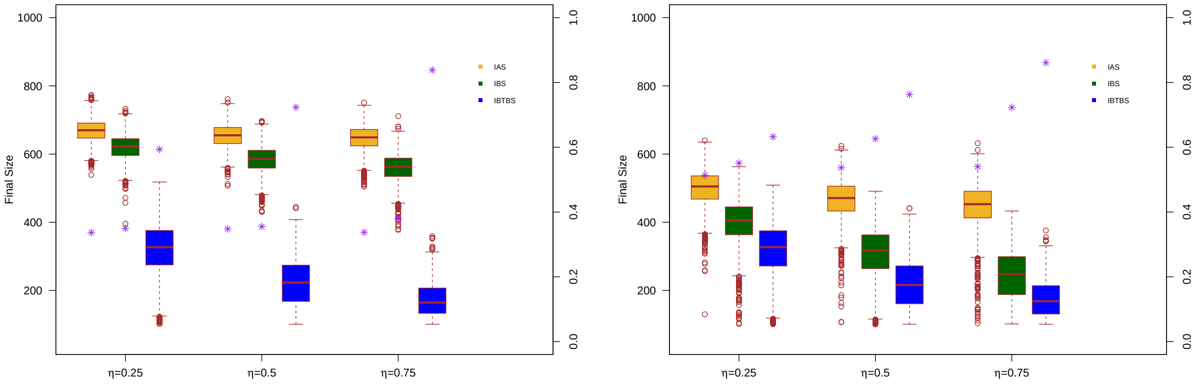

Figure S8. Distributions of the final size value for Scenario IAS (yellow), Scenario IBS (green) and Scenario IBTBS (blue) when the quarantine contact rate is λ_q=0.5λ (left panel) and λ_q=0.1λ (right panel) together with the probability that a simulation leads to a number of cases smaller than the 10% of the population (purple asterisks)

Reproduction Number

In Figure S9 and S10, we vary the reproduction number of symptomatic individuals that is set, respectively, to $\mathcal{R}_0^s = 2$ and $\mathcal{R}_0^s = 3$. We assume that also in these cases the asymptomatic reproduction number is equal to $\mathcal{R}_0^s = 0.55\mathcal{R}_0^s$. Therefore, this is set, respectively, to $\mathcal{R}_0^a = 1.1$ and $\mathcal{R}_0^a = 1.65$. The effect of the antiviral drug, in addition to isolation and quarantine, increases when the reproduction number increases. In case of $\mathcal{R}_0 = 3$, the peak incidence decreases significantly when the antiviral compound is used, compared to control measures based only on isolation and quarantine after symptoms onset. In Figure S11 we set asymptomatic and symptomatic individuals to have the same reproduction number of value $\mathcal{R}_0 = 2.5$. Also in this scenario we noticed that the administration of an antiviral drug lead to a substantial decrease in the final size and peak incidence compared to the values obtained when the other control measures are considered.

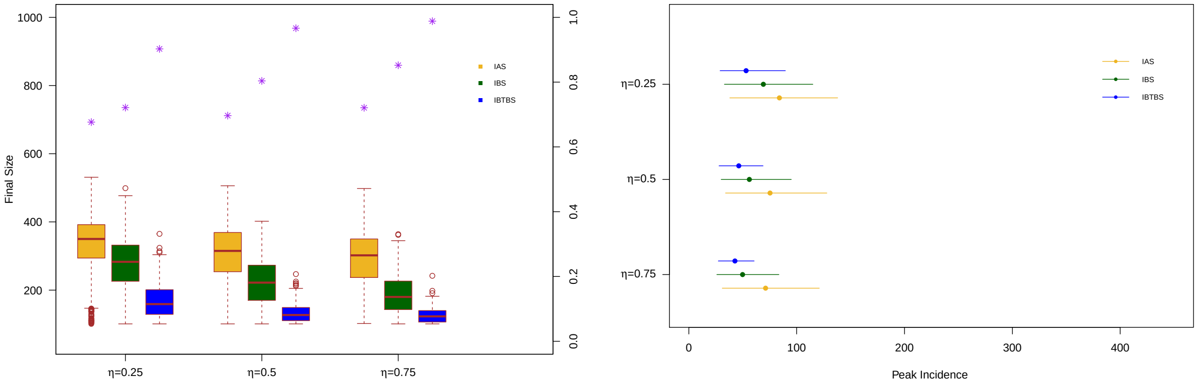

Figure S9. Final size distribution (left panel) and mean peak incidence (right panel) for Scenario IAS (yellow), Scenario IBS (green) and Scenario IBTBS (blue) when the quarantine contact rate is λ_q=0.25λ, $\mathcal{R}_0^s = 2$ and $\mathcal{R}_0^a = 0.55\mathcal{R}_0^s$. In the left panel, for each scenario we report the probability that a simulation leads to a number of cases smaller than the 10% of the population (purple asterisks). In the right panel, together with the point estimates we report the 2.5% and 97.5 % percentiles.

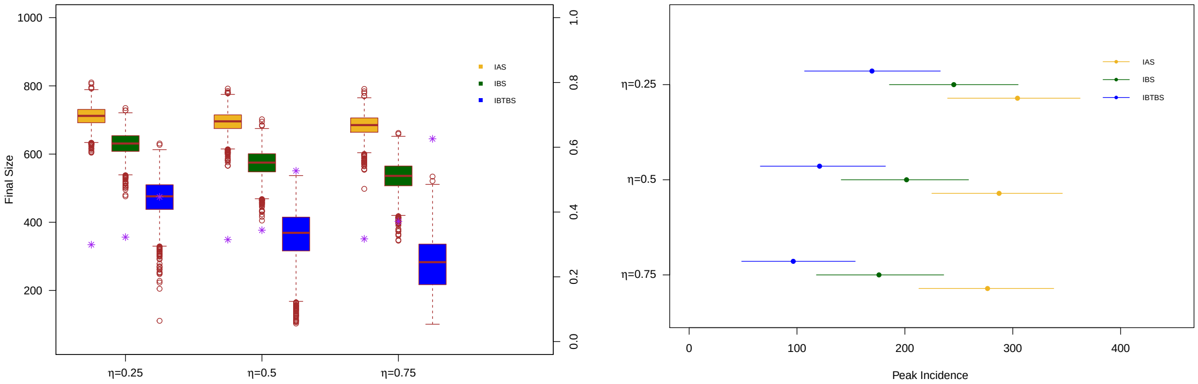

Figure S10. Final size distribution (left panel) and mean peak incidence (right panel) for Scenario IAS (yellow), Scenario IBS (green) and Scenario IBTBS (blue) when the quarantine contact rate is λ_q=0.25λ, $\mathcal{R}_0^s = 3$ and $\mathcal{R}_0^a = 0.55\mathcal{R}_0^s$. In the left panel, for each scenario we report the probability that a simulation leads to a number of cases smaller than the 10% of the population (purple asterisks). In the right panel, together with the point estimates we report the 2.5% and 97.5 % percentiles.

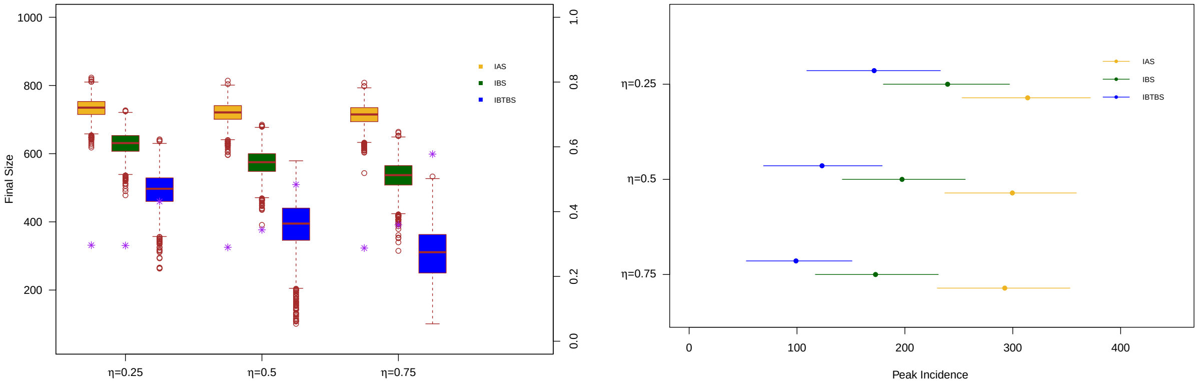

Figure S11. Final size distribution (left panel) and mean peak incidence (right panel) for Scenario IAS (yellow), Scenario IBS (green) and Scenario IBTBS (blue) when the quarantine contact rate is λ_q=0.25 λ. We consider the same reproduction number for asymptomatic ($\mathcal{R}_0^a$) and symptomatic ($\mathcal{R}_0^s$) individuals: $\mathcal{R}_0^a = \mathcal{R}_0^s = 2.5$. In the left panel, for each scenario we report the probability that a simulation leads to a number of cases smaller than the 10% of the population (purple asterisks). In the right panel, together with the point estimates we report the 2.5% and 97.5 % percentiles.

Test detection period

In Figure S12 and Figure S13 we report the effect of a longer time needed for the test to detect an infectious individual. We assume the test is positive when performed on an infectious individual who has been infected since at least 3 or 4 days. The traced individuals who result negative, are tested again after, respectively, 3 or 4 days. Simulations show a substantial increase, both in the final size and the peak incidence for the IBS scenario. Instead, the use of an antiviral drug results also in this case of remarkable impact in both the final size and the peak incidence.

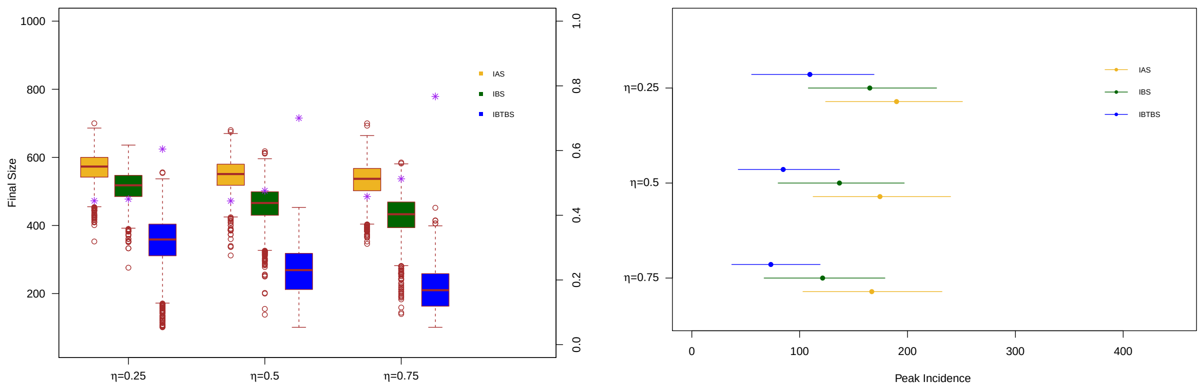

Figure S12. Final size distribution (left panel) and mean peak incidence (right panel) for Scenario IAS (yellow), Scenario IBS (green) and Scenario IBTBS (blue) when the quarantine contact rate is λ_q=0.25 λ, $\mathcal{R}_0^s$=2.5, $\mathcal{R}_0^a$=0.55$\mathcal{R}_0^s$ and the test detect positively an infectious individual after 3 days since infection. In the left panel, for each scenario we report the probability that a simulation leads to a number of cases smaller than the 10% of the population (purple asterisks). In the right panel, together with the point estimates we report the 2.5% and 97.5 % percentiles.

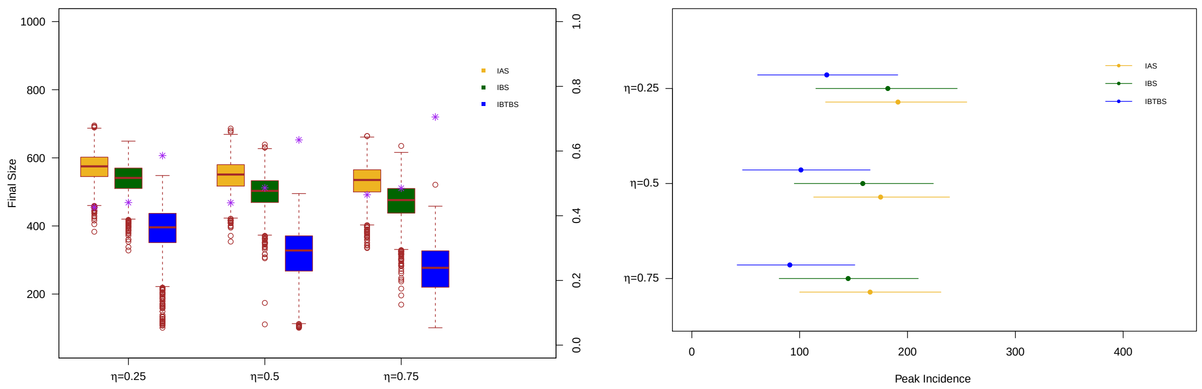

Figure S13. Final size distribution (left panel) and mean peak incidence (right panel) for Scenario IAS (yellow), Scenario IBS (green) and Scenario IBTBS (blue) when the quarantine contact rate is λ_q=0.25 λ, $\mathcal{R}_0^s$=2.5, $\mathcal{R}_0^a$=0.55$\mathcal{R}_0^s$ and the test detect positively an infectious individual after 4 days since infection. In the left panel, for each scenario we report the probability that a simulation leads to a number of cases smaller than the 10% of the population (purple asterisks). In the right panel, together with the point estimates we report the 2.5% and 97.5 % percentiles.

Asymptomatic proportion

In addition to the baseline scenario in which 31% of infectives are asymptomatic, we tested the effect of different asymptomatic proportions. In Figure S14 and S15 the symptomatic proportion is set, respectively, to 100% and 14%. The former scenario represent the case in which all individuals shows symptoms and are diagnosed while the former is set to represent the proportion of undiagnosed individuals reported in Li et al. [4] that includes asympyomatic and mild symptomatic individuals. We noticed that the administration of an antiviral drug lead to a substantial decrease in the final size and peak incidence when the proportion of symptomatic and consequently diagnosed is high, compared to the other considered control measures. The efficacy of the control measure based on antiviral decreases when a high proportion of individuals are asymptomatic remaining, however, the containment strategy that performs better.

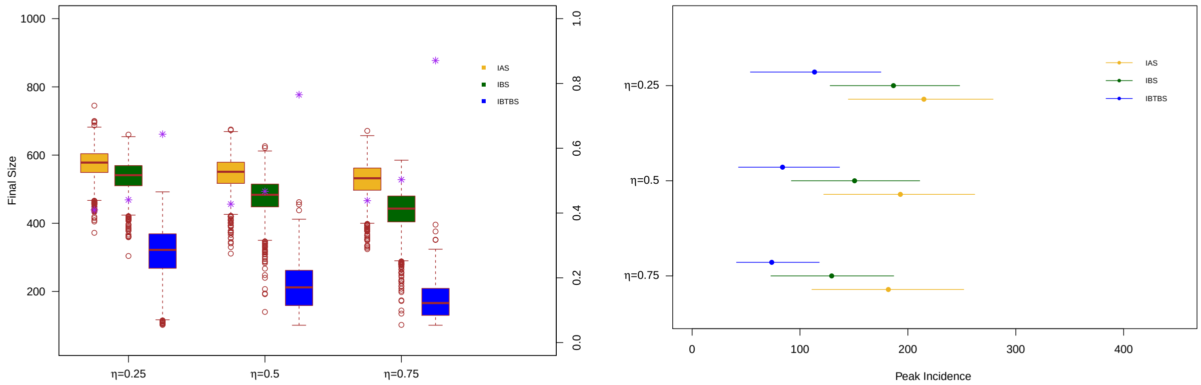

Figure S14. Final size distribution (left panel) and mean peak incidence (right panel) for Scenario IAS (yellow), Scenario IBS (green) and Scenario IBTBS (blue) when the quarantine contact rate is λ_q=0.25 λ, and the proportion of symptomatic individuals is 100%. The reproduction number of symptomatic cases is set to $\mathcal{R}_0^s = 2.5$. In the left panel, for each scenario we report the probability that a simulation leads to a number of cases smaller than the 10% of the population (purple asterisks). In the right panel, together with the point estimates we report the 2.5% and 97.5 % percentiles.

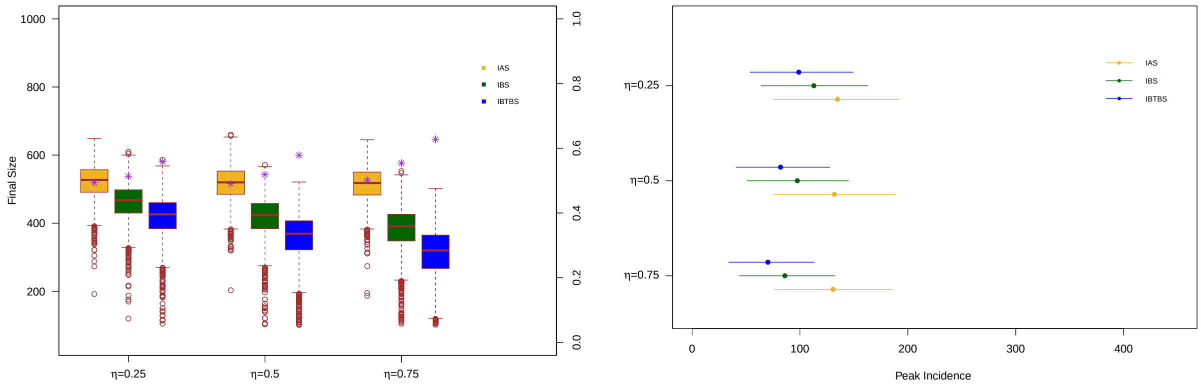

Figure S15. Final size distribution (left panel) and mean peak incidence (right panel) for Scenario IAS (yellow), Scenario IBS (green) and Scenario IBTBS (blue) when the quarantine contact rate is λ_q=0.25 λ, and the proportion of symptomatic individuals is 14%. The reproduction number of symptomatic cases is set to $\mathcal{R}_0^s = 2.5$ while $\mathcal{R}_0^a = 0.55\mathcal{R}_0^s$. In the left panel, for each scenario we report the probability that a simulation leads to a number of cases smaller than the 10% of the population (purple asterisks). In the right panel, together with the point estimates we report the 2.5% and 97.5 % percentiles.

Initial infectives

We checked the impact of the initial number of infectives who start the outbreak. We noticed that final size and peak incidence are the same as in the baseline scenario. Instead, the probability that the outbreak will fade out decreases when more initial infectives are considered. However, for a control measure based on antiviral, the fade out probability is remarkably higher compare to the other tested strategies, with a value greater than 0.5 when the contact tracing has an high probability of success (Figure S16).

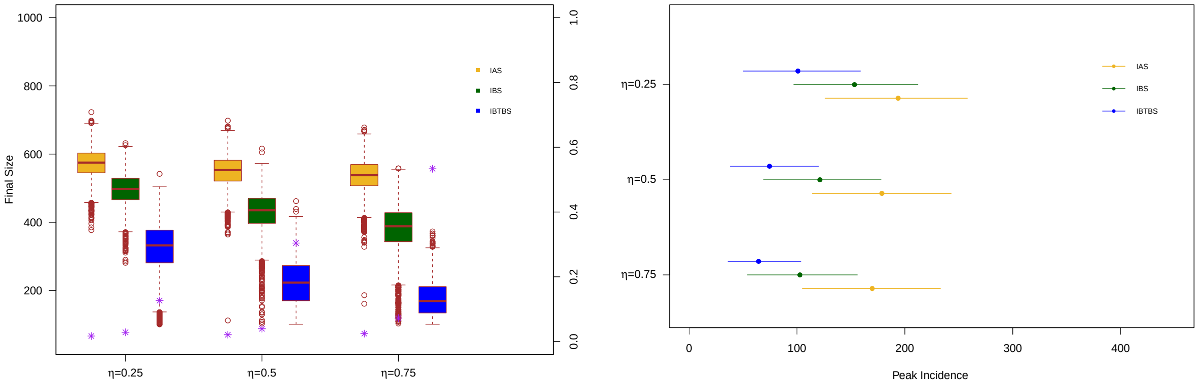

Figure S16. Final size distribution (left panel) and mean peak incidence (right panel) for Scenario IAS (yellow), Scenario IBS (green) and Scenario IBTBS (blue) when the outbreak starts with 5 initial infectives. The quarantine contact rate is λ_q=0.25 λ, the reproduction number of symptomatic cases is set to $\mathcal{R}_0^s = 2.5$ while $\mathcal{R}_0^a = 0.55\mathcal{R}_0^s$. In the left panel, for each scenario we report the probability that a simulation leads to a number of cases smaller than the 10% of the population (purple asterisks). In the right panel, together with the point estimates we report the 2.5% and 97.5 % percentiles.

References

[1] Gompertz B. Xxiv. On the nature of the function expressive of the law of human mortality, and on a new mode of determining the value of life contingencies. In a letter to Francis Baily, Esq. FRS &c. Philosophical transactions of the Royal Society of London. 1825;(115):513–583.

[2] Sheahan TP, Sims AC, Leist SR, Schäfer A, Won J, Brown AJ, et al. Comparative therapeutic efficacy of remdesivir and combination lopinavir, ritonavir, and interferon beta against MERS-CoV. Nature Communications. 2020;11(1):222. Available from: `https://doi.org/10.1038/s41467-019-13940-6`.

[3] Backer JA, Klinkenberg D, Wallinga J. Incubation period of 2019 novel coronavirus (2019-nCoV) infections among travellers from Wuhan, China, 20–28 January 2020. Eurosurveillance. 2020;25(5).

[4] Li R, Pei S, Chen B, Song Y, Zhang T, Yang W, et al. Substantial undocumented infection facilitates the rapid dissemination of novel coronavirus (SARS-CoV2). Science. 2020;.
